# A review of non-microbial biological control strategies against the Asian longhorned beetle (Coleoptera: Cerambycidae)

**DOI:** 10.1093/ee/nvae116

**Published:** 2024-11-20

**Authors:** Courtney L Johnson, David R Coyle, Jian J Duan, Seunghwan Lee, Seunghyun Lee, Xiaoyi Wang, Xingeng Wang, Kelly L F Oten

**Affiliations:** Department of Forestry and Environmental Resources, North Carolina State University, Raleigh, NC, USA; Department of Forestry and Environmental Conservation, Clemson University, Clemson, SC, USA; Beneficial Insects Introduction Research Unit, Agricultural Research Service, United States Department of Agriculture, Newark, DE, USA; Department of Agricultural Biotechnology, College of Agriculture and Life Sciences, Seoul National University, Seoul, South Korea; Department of Life Sciences, Natural History Museum, London, UK; Key Laboratory of Forest Protection of National Forestry and Grassland Administration, Ecology and Nature Conservation Institute, Chinese Academy of Forestry, Beijing, China; Beneficial Insects Introduction Research Unit, Agricultural Research Service, United States Department of Agriculture, Newark, DE, USA; Department of Forestry and Environmental Resources, North Carolina State University, Raleigh, NC, USA

**Keywords:** forest health, forest pest, invasive species, management, *Ontsira*, parasitoid

## Abstract

The Asian longhorned beetle (ALB), *Anoplophora glabripennis* (Motschulsky), is a polyphagous woodboring beetle that infests and damages hardwood host trees in Asia, Europe, and North America. Native to China and the Korean peninsula, ALB is invasive in both North America and Europe. Due to the large environmental and economic impacts associated with ALB, much effort has been placed on its management and eradication from invaded areas. Eradication programs consist of visual surveys, regulatory quarantines, host removal, public outreach and education, and in some cases, insecticides. Host removal is effective but is laborious and costly, and while insecticides have been useful as a component of some eradication programs, they can be expensive, ineffective, and environmentally detrimental. Thus, several arthropod biological control agents (BCAs) have been evaluated which could support a more environmentally friendly management strategy to supplement traditional ALB management tactics. Here, we review the biological control strategy for ALB, including the exploration within the native and invaded ranges of the pest, to find potential arthropod BCAs. We discuss the ecological premise behind the method as well as the potential for its success, and we identify knowledge gaps and future considerations for the enactment of this method. While biological control shows promise, care will be needed in utilizing this method, and further research must explore the success of BCAs in field settings.

## Biological Control: Ecological Premises and Perspectives for Success

Many non-native insect species achieve invasive pest status when they are accidentally transported to new locations where local biotic and abiotic factors are ineffective in suppressing their populations and coevolved natural enemy complexes are lacking. It is generally agreed that some of the most effective natural enemies of an exotic pest are those that have coevolved with it in its native range (van den Bosch et al. 1982). Therefore, some of the most dramatic successes in biological control have resulted from the introduction of natural enemies from the target pest’s native range, termed classical biological control (see reviews in Clausen et al. 1978, Van Driesche and Bellows 1996, Hoddle 2003, and Van Driesche et al. 2010). A high rate (> 62%) of success in classical biological control projects (conducted prior to 2010) against invasive arthropod pests has been demonstrated in North American forests (Van Driesche et al. 2010, 2020).

Introduced pests may also be controlled through novel-association biological control. Novel-association biological control originally referred to the use of exotic natural enemies for the control of native pests (Hokkanen and Pimentel 1989) but also applies to the situations when indigenous parasitoids have adapted to exotic hosts. Novel associations in parasitoids could arise from phylogenetic relatedness in detectability and suitability between coevolved and novel hosts, whereby parasitoids can use a suite of evolved traits from an old association to explore novel hosts that are ecologically and physiologically similar to their ancestral hosts (Hokkanen and Pimentel 1989, Duan et al. 2024). The biological characteristics of ectoparasitoids could make them very suitable novel association BCAs because they typically do not need to circumvent host internal defenses (see Wang et al. 2021c). These larval parasitoids typically locate hosts using a probable combination of surface vibrational cues and chemical cues perceived when the host is stung (Paine 2017), rendering previously attacked (paralyzed) hosts no longer detectable by other parasitoids. In doing so, they reduce superparasitism or multi-parasitism and promote coexistence and synergistic regulation of host populations by different larval parasitoids (Wang and Aparicio 2020, Wang et al. 2021c)

In their native range, population dynamics of phloem- and woodboring beetles are often regulated by a complex of abiotic (e.g., weather) and biotic factors such as host tree resistance, interspecific competition, and co-adapted natural enemies. Although evidence that the population dynamics of phloem- or woodboring beetles are solely regulated by natural enemies is scant, populations of an exotic cerambycid, *Phoracantha semipunctata* (Fabricius) have been successfully controlled by several species of introduced egg and larval parasitoids in California, United States (Hanks et al. 1996, 2001). Additionally, in China, control of native cerambycid pests such as *Monochamus alternatus* Hope and *Massicus raddei* (Blessig) has been demonstrated in the field with several parasitoids (Yang et al. 2014). More recently, 2 introduced larval parasitoids, *Tetrastichus planipennisi* Yang (Hymenoptera: Eulophidae) and *Spathius galinae* Belokobylskij and Strazanac (Hymenoptera: Braconidae) appeared to have successfully reduced the population growth of the invasive emerald ash borer, *Agrilus planipennis* Fairmaire (Coleoptera: Buprestidae) (EAB), in Michigan and several northeastern states (Duan et al. 2021, 2022, 2023). These examples of success illuminate the underlying ecological principles of biological control and demonstrate that it can be an effective management strategy against invasive phloem- and woodboring beetles.

## Asian Longhorned Beetle

**Fig. 1.**
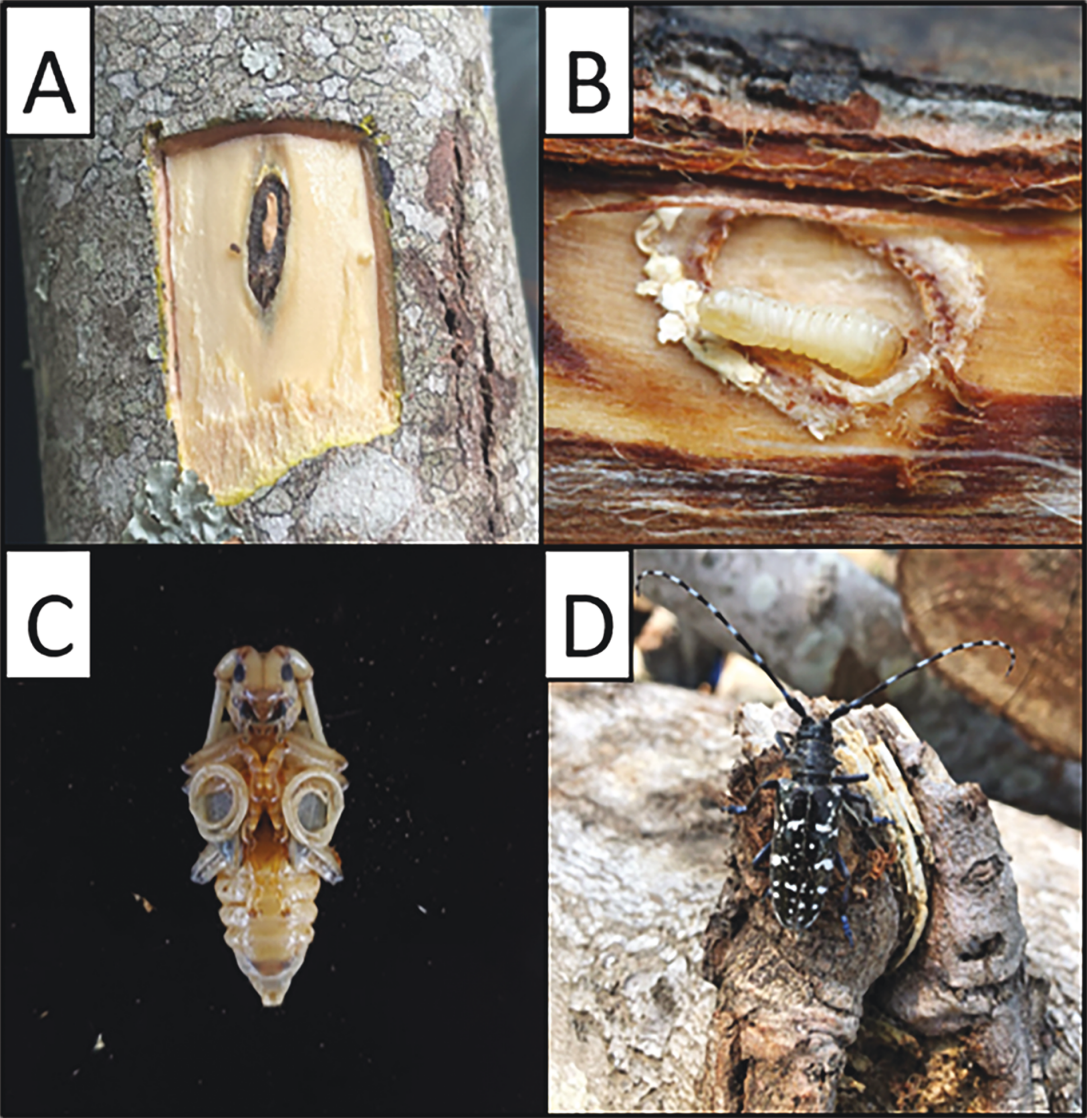
The life stages of ALB, egg (A), larva (B), pupa (C), and adult (D). Photo credits: (A) Lindsey Stone, Clemson University; (B) Seunghyun Lee, Natural History Museum London; (C) Abby Ratcliff, North Carolina State University; (D) David Coyle, Clemson University

**Fig. 2.**
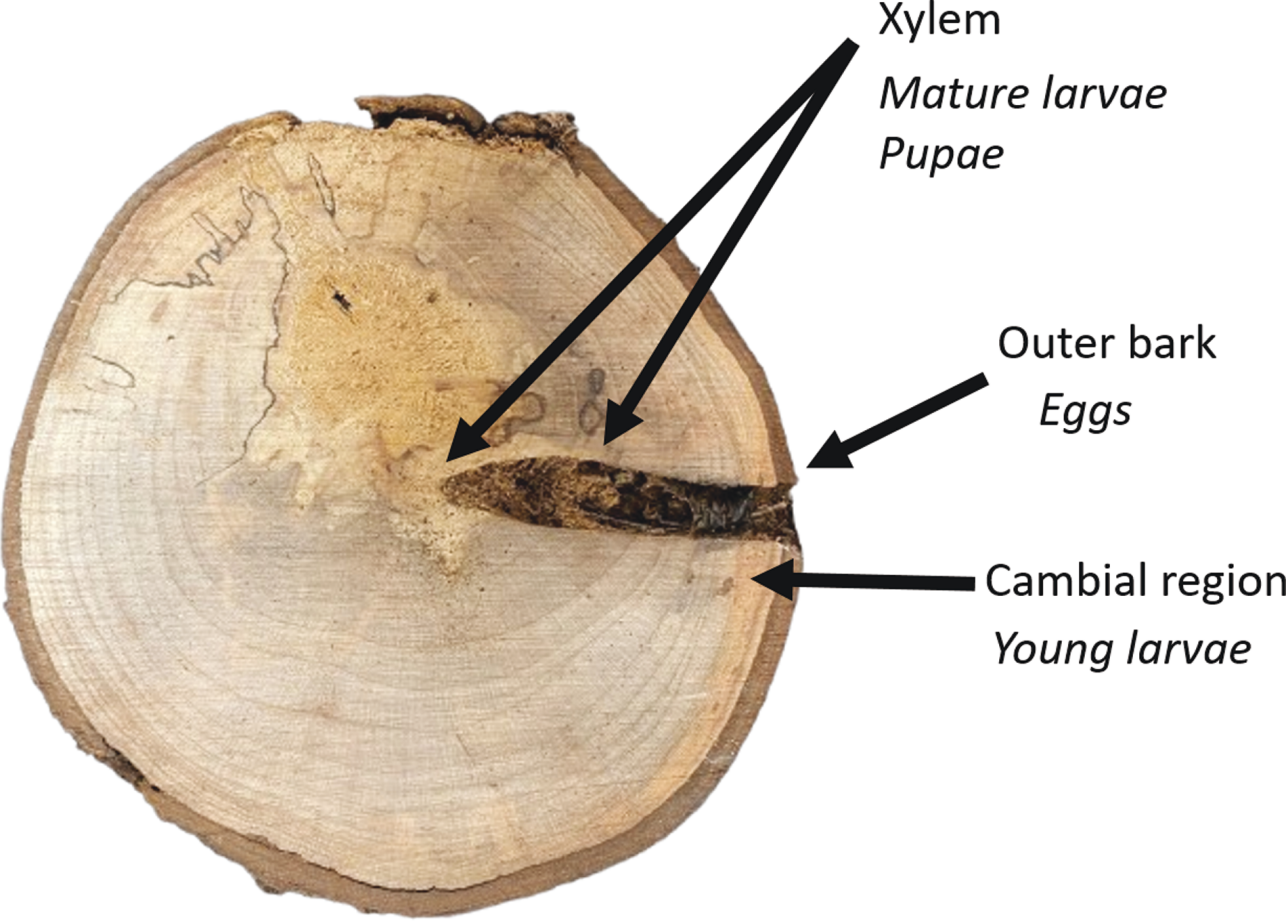
A cross-section of wood with ALB damage. Host tree tissues are labeled at the end of arrows, and ALB life stages occurring within these tissues are labeled underneath in italics. Young larvae are those in the first through third instars, and mature larvae are those in the fourth instar and beyond.

The Asian longhorned beetle (ALB), *Anoplophora glabripennis* (Motschulsky) (Coleoptera: Cerambycidae), is an invasive woodboring beetle that has caused extensive tree mortality in Asia, North America, and Europe. While all *Anoplophora* species are native to Asia, the native range of ALB includes only China and the Korean peninsula (Wu and Chiang 1998, Lingafelter and Hoebeke 2002, Williams et al. 2004, Hu et al. 2009, Haack et al. 2010). The beetle has spread inadvertently due to infested wood packaging material used in commodity movement (Haack et al. 2010), and it is now an invasive pest in both North America and Europe (Table 1). The beetle is also pestiferous in parts of its native range. For instance, in South Korea, ALB has been reported in urban areas as a pest of *Aesculus turbinata* Blume (Sapindales: Hippocastanaceae), *Salix koreensis* Anderson (Salicales: Salicaceae), and *Ulmus parvifolia* Jacq. (Urticales: Ulmaceae) (Lee et al. 2020), and in China, it has a wide distribution across 29 provincial administrative regions and is considered a pest primarily of poplar (*Populus* spp.) plantations and urban plantings (Sun et al. 2019). While ALB can use 23 genera of hardwood trees as hosts (EPPO 2023), it strongly prefers *Acer* spp. in both its native and invaded ranges (Haack et al. 2010, Coyle et al. 2021, Turgeon et al. 2022). Early ALB instars feed within the phloem and cambium; later instars tunnel into the sapwood and heartwood, reducing water and nutrient flow and weakening structural integrity, ultimately resulting in tree death (Figs. 1 and 2).

**Table 1.** Invasion history of ALB summarized by country, year recorded, and year declared eradicated

Country	Year first recorded	Year declared eradicated	References
United States	1996	Active * * Eradicated in 2008 in Illinois and 2013 in New Jersey. Active infestations still occur within the United States in parts of Ohio, New York, Massachusetts, and South Carolina.	Haack et al. 1997, EPPO 2023, USDA-APHIS 2024
Austria	2001	2021	EPPO 2023
Japan	2002	Active * * Eradicated in 2003, but a new infestation was discovered in 2021.	Akita et al. 2021, EPPO 2023
Canada	2003	2020 * *Declared eradicated in April 2013, but a satellite infestation was discovered in September 2013. The satellite infestation was eradicated by 2020.	Turgeon et al. 2015, Turgeon et al. 2022, EPPO 2023
France	2003	Active * * Eradicated from Strasbourg in 2019 and Corsica in 2022. Active infestations still occur within France.	EPPO 2023
Italy	2007	Active * *Eradicated from Veneto in 2020. Active infestations still occur within Italy.	EPPO 2023
Belgium	2009	2011	EPPO 2023
Netherlands	2010	2016	EPPO 2023
Switzerland	2011	Active * *Eradicated in 2020, but a new outbreak was detected in 2022.	EPPO 2023
United Kingdom	2012	2019	EPPO 2023
Finland	2015	2021	EPPO 2023
Montenegro	2015	2020	EPPO 2023
South Korea	2020	Active * *While ALB is native to South Korea, an invasive urban population was discovered through genetic analysis.	Lee et al. 2020

In some noted instances, ALB has been eradicated from part of a country while active infestations still occur in other parts.

Eradication of ALB has occurred in about 50% of establishments worldwide (Branco et al. 2022, Turgeon et al. 2022, Wang et al. 2023; Table 1). While they differ by location, there are commonalities among eradication programs, including the incorporation of regulated zones, surveys, and public outreach (Smith et al. 2009, Branco et al. 2022, Turgeon et al. 2022). Host removal is the most common eradication tactic used (Branco et al. 2022), which typically involves removing trees, chipping them, and grinding stumps (Smith et al. 2009, Haack et al. 2010, Meng et al. 2015). In addition to the mechanical removal of host trees, chemical treatment has been used to augment host removal in Asia and North America but is not commonly used in Europe (Branco et al. 2022). For example, imidacloprid was used for control in Chicago, United States, and cypermethrin and aluminum phosphide have been utilized in China (Hu et al. 2009, Haack et al. 2010, Wang et al. 2023). Lastly, planting trap trees, utilizing the “push and pull” method (planting host species alongside repellent species like *Melia azedarach* Linnaeus [Sapindales: Meliaceae]), and replacing poplar monocultures with a diversity of tree species also aid ALB management or help slow the spread (Hu et al. 2009, Wang et al. 2023).

While host tree removal is successful in achieving eradication, it is costly and takes years to achieve. Similarly, insecticides are only marginally effective and are not feasible at a large scale due to cost and because systemic chemicals (those that are applied and taken up in the tree’s vascular system) typically only affect young larvae in the cambial region and outer sapwood (Poland et al. 2006). Thus, effective biological control agents (BCAs) could provide another tool to supplement these tactics and reduce ALB populations. This includes the use of microbial pesticides, the attraction of entomophagous birds (such as woodpeckers), and the release of arthropod natural enemies (Lacey et al. 2015). In this article, we review biological control survey efforts and potential BCAs that have been discovered for the control of ALB. We focus on arthropod predators and parasitoids and vertebrate predators, as Hajek et al. (2024) provide a recent review of microbial BCAs for ALB. Lastly, we assess the potential for success in using biological control, reflect on current gaps in knowledge, and discuss important considerations for the future incorporation of BCAs into an ALB management strategy.

## Potential Biological Control Agents

### Predators

While much focus is placed on parasitoids for biological control, predators are also important in reducing pest populations. Various arthropod species, including coleopteran (e.g., Cleridae, Elateridae, Histeridae, Melyridae), raphidiid (Raphidiidae, Inoceliidae), dipteran (e.g., Asilidae, Pallopteridae), and dermapteran predators are often found in the galleries of woodboring beetles (see Kenis and Hilszczanski 2007 and references therein). These polyphagous predators may prey upon larvae or pupae of any cerambycid beetles, though their predatory impacts have been documented in only a few cases, and we know of no studies that have documented their impact on ALB in its invaded regions. For example, Pulkkinen and Yang (1984) showed that the dipteran odiniid *Odinia xanthocera* Collin killed about 10.5% of the population of *Saperda populnea* (Linnaeus) (Coleoptera: Cerambycidae) in Finland. Like ALB larvae, *S. populnea* larvae create oval feeding tunnels and push some of the woody debris and frass out of the tunnel. This fly lays eggs at the entrance of the host tunnel, and the newly hatched larvae enter the tunnel to find and feed on the beetle pupae.

In addition to arthropod natural enemies, vertebrate predators may play a role in reducing cerambycid populations. Woodpeckers are the most important predators of various woodboring beetles (Yang 2005, Kenis and Hilszczanski 2007, Paine 2017), and cerambycid larvae can be an important food for some woodpecker species While no studies to date have documented impacts of woodpeckers on ALB in its invaded range, in China, several studies have documented significant impacts of 2 woodpeckers, *Dendrocopos major* (Linnaeus) and *Picus canus* Gmelin (both Piciformes: Picidae), on ALB or other closely related cerambycids in several areas in Ningxia, Gansu, Henan, Inner Mongolia, and Tianjin (Yang 2005). Zhang (1986) reported that 29-58% of *D. major*’s diet consisted of cerambycid larvae during the brood time when each pair of woodpeckers could consume as many as 2,500 larvae. These studies were conducted to encourage the nesting of woodpeckers in ALB-infested forests by planting mixed forests, increasing forest coverage, conserving deadwood, and providing nests (e.g., Li et al. 2001, Hao et al. 2003). In a poplar forest in Inner Mongolia, Hao et al. (2003) observed that a pair of *D. major* adults laid 3–5 eggs, and 70–80% of these eggs hatched in May. The adults’ predation area ranged from 20–40 hectares, and authors found that 8% of the poplar trees had natural nests. In poplar logs, 62% of sentinel ALB larvae were preyed upon by *D. major*, and authors estimated that *D. major* could effectively control about 25 hectares of forest per pair. Woodpeckers have become the dominant natural enemy against borer pests in Tianjin where the average predatory rate reached 22% (Liu et al. 1995). Notably, a field study from 1996 to 1998 showed that both *P. canus* and *D. major* were dominant predatory birds of *Anoplophora nobilis* Ganglbauer (this species was ALB but reported as the former yellow spotted biotype of ALB; see Lingafelter and Hoebeke 2002) in Longxi County, Gansu Province where 54–60% of larvae and eggs on the sampled trees were attacked by these birds in a 209-hectare *Populus* forest (Zhu 2002). Consequently, the average population density of *A. nobilis* larvae was reduced from 16 per tree to 3 per tree over 3 yr. These studies showed that woodpeckers can provide effective control of cerambycids in some forests. However, woodpeckers may also prey on parasitoid larvae and cocoons (Schimitschek 1929) or further weaken the main branches already damaged by beetles (Pulkkinen and Yang 1984).

So far, predators have been rarely used in classical or augmentative biological control of ALB but have the potential for conservation biocontrol as shown in these studies. More often, parasitoids are utilized for biological control efforts.

### Parasitoids

To date, 29 species of parasitoids from 2 insect orders and seven families are known to attack ALB (Table 2). This list is based largely on several recent explorations for ALB parasitoids in China (Liu et al. 2016, Golec et al. 2018, Li et al. 2020, Wei et al. 2023) and Korea (Kim et al. 2018, Lee et al. 2023), as well as surveys and laboratory tests of indigenous parasitoids in Italy (Hérard et al. 2013, Brabbs et al. 2014, Lupi et al. 2017) and the United States (Duan et al. 2016, Golec et al. 2020). Prior to these surveys, few parasitic wasps were known to attack ALB. These explorations have provided potential BCAs for ALB management, both through classical biological control and novel association biological control.

**Table 2.** List of known species of parasitoids worldwide capable of using *Anoplophora glabripennis* as a host

Order	Family	Species	Country	Host stage	Mode of parasitism			References
Coleoptera	Bothrideridae	*Dastarcus helophoroides* (Sharp)	China	Larva/pupa	Gregarious	Ecotoparasitoid	Idiobiont	Haack et al. 2010, Liu et al. 2016
Hymenoptera	Bethylidae	*Sclerodermus guani* (Xiao et Wu) *	China	Larva	Gregarious	Ecotoparasitoid	Idiobiont	Liu et al. 2016, Li et al. 2020, Wei et al*.* 2023
Hymenoptera	Bethylidae	*Sclerodermus sichuanensis* Xiao	China	Larva	Gregarious	Ecotoparasitoid	Idiobiont	Haack et al. 2010
Hymenoptera	Bethylidae	*Sclerodermus* sp.	China	Young larva	Gregarious	Ecotoparasitoid	Idiobiont	Li et al. 2020
Hymenoptera	Bethylidae	*Sclerodermus brevicornis* (Kieffer)	Italy	Larva	Gregarious	Ecotoparasitoid	Idiobiont	Lupi et al. 2017
Hymenoptera	Braconidae	*Atanycolus* spp.	USA	Young larva	Gregarious	Ecotoparasitoid	Idiobiont	Duan et al. 2016
Hymenoptera	Braconidae	*Bracon planitibiae* Yang *	China	Young larva	Gregarious	Ecotoparasitoid	Idiobiont	Li et al. 2020, Wei et al. 2023
Hymenoptera	Braconidae	*Heterospilus* spp.	USA	Young larva	Gregarious	Ecotoparasitoid	Idiobiont	Duan et al. 2016
Hymenoptera	Braconidae	*Leluthia honshuensis* Belokobylskij & Maeto *	South Korea	Young larva	Gregarious	Ecotoparasitoid	Idiobiont	Kim et al. 2018
Hymenoptera	Braconidae	*Ontsira mellipes* Ashmead	USA	Young larva	Gregarious	Ecotoparasitoid	Idiobiont	Duan et al. 2016
Hymenoptera	Braconidae	*Rhoptrocentrus piceus* Marsh	USA	Young larva	Gregarious	Ecotoparasitoid	Idiobiont	Duan et al. 2016
Hymenoptera	Braconidae	*Spathius anoplophorae* sp. nov. Yang *	China	Young larva	Gregarious	Ecotoparasitoid	Idiobiont	Li et al. 2020, Wei et al. 2023
Hymenoptera	Braconidae	*Spathius erythrocephalus* Wesmael	Italy	Young larva	Gregarious	Ecotoparasitoid	Idiobiont	Hérard et al. 2013
Hymenoptera	Braconidae	*Spathius ibarakius* Belokobylskij & Maetô *	South Korea	Young larva	Gregarious	Ecotoparasitoid	Idiobiont	Lee et al. 2023
Hymenoptera	Braconidae	*Spathius laflammei* Provancher	USA	Young larva	Gregarious	Ecotoparasitoid	Idiobiont	Duan et al. 2016
Hymenoptera	Braconidae	*Zombrus bicolor* (Enderlein)	China	Young larva	Solitary	Ecotoparasitoid	Idiobiont	Li et al. 2020
Hymenoptera	Eupelmidae	*Calosota agrili* Curtis	Italy	Young larva	Gregarious	Ecotoparasitoid	Idiobiont	Hérard et al. 2013
Hymenoptera	Eupelmidae	*Eupelmus aloysii* Russo	Italy	Young larva	Gregarious	Ecotoparasitoid	Idiobiont	Hérard et al. 2013
Hymenoptera	Eupelmidae	*Eupelmus urozonus* Dalman *	China	Young larva	Gregarious	Ecotoparasitoid	Idiobiont	Li et al. 2020
Hymenoptera	Eurytomidae	*Eurytoma melanoneura* Walker	Italy	Young larva	Gregarious	Ecotoparasitoid	Idiobiont	Hérard et al. 2013
Hymenoptera	Eurytomidae	*Eurytoma morio* Boheman	Italy	Young larva	Solitary	Ecotoparasitoid	Idiobiont	Hérard et al. 2013
Hymenoptera	Eurytomidae	*Eurytoma chinensis* Yang *	China	Young larva	Solitary	Ecotoparasitoid	Idiobiont	Wei et al. 2023
Hymenoptera	Ichneumonidae	*Iphiaulax imposter* (Scopoli)	China	Young larva	Gregarious	Ecotoparasitoid	Idiobiont	Tang et al. 1996
Hymenoptera	Pteromalidae	*Cleonymus brevis* Boucek	Italy	Young larva	Gregarious	Ecotoparasitoid	Idiobiont	Hérard et al. 2013
Hymenoptera	Pteromalidae	*Oxysychus glabripennis* Yang *	China	Young larva	Gregarious	Ecotoparasitoid	Idiobiont	Li et al. 2020, Wei et al. 2023
Hymenoptera	Pteromalidae	*Trigonoderus princeps* Westwood	Italy	Young larva	Gregarious	Ecotoparasitoid	Idiobiont	Hérard et al. 2013
Hymenoptera	Pteromalidae	*Zolotarewskya anoplophora* sp. nov. Yang *	China	Young larva	Gregarious	Ecotoparasitoid	Idiobiont	Li et al. 2020, Wei et al. 2023
Hymenoptera	Pteromalidae	*Callocleonymus beijingensis* Yang *	China	Young larva	Gregarious	Ecotoparasitoid	Idiobiont	Li et al. 2020
Hymenoptera	Pteromalidae	*Zolotarewskya microdentata* Yang	China	Young larva	Gregarious	Ecotoparasitoid	Idiobiont	Li et al. 2020, Wei et al. 2023

Those discovered from sentinel bolt surveys are denoted with an asterisk.

### Potential for Classical Biological Control: Parasitoids of ALB in Asia

Investigations of ALB natural enemies native to Asia have primarily been carried out within the outbreak populations associated with monoculture plantations of susceptible host trees in China (e.g., Wang et al. 1999, Hu et al. 2009, Yang et al. 2014, Liu et al. 2016, Golec et al. 2018). These explorations have discovered parasitoids including *Iphiaulax imposter* (Scopoli) (Hymenoptera: Braconidae) (Tang et al. 1996, Gao and Li 2001). *Iphiaulax imposter* was found in Yinchuan city and Qingtongxia city of Ningxia Province, and it has a natural parasitism rate of around 4%, with peak periods for pupation and emergence of adults occurring in late May and mid-to-late June, respectively. Perhaps two of the most widely used BCAs within the native range of ALB are *Dastarcus helophoroides* Fairmaire (Coleoptera: Bothrideridae) and *Sclerodermus* spp. (Hymenoptera: Bethylidae).

#### Dastarcus helophoroides

*Dastarcus helophoroides* primarily targets the mature larvae, prepupae, and pupae of cerambycids and has been regarded as a dominant natural enemy of ALB in its native range (Zhou et al. 1985, Li et al. 2009, Wei and Niu 2011). Rather than ovipositing directly on the host, *D. helophoroides* oviposits on the bark, within-host galleries, or in frass; neonates must then navigate host galleries in search of a host larva before it tunnels deeper into the wood and becomes inaccessible (Qin and Gao 1988, Lei et al. 2003). The application of *D. helophoroides* in augmentative biological control programs has succeeded in China, South Korea, and Japan (Luo et al. 2021). One of the earliest records of using *D. helophoroides* against ALB in China was reported by Zhou et al. (1985) in Tianshui City, Gansu Province. *Dastarcus helophoroides* was released on the trunk of ALB-infested trees, and ALB emergence rates decreased significantly where the parasitoid was released (9.1% compared to 62.4% on control) (Zhou et al. 1985). Subsequent studies by Li et al. (2009) demonstrated that newly hatched *D. helophoroides* larvae exhibited the ability to locate and parasitize ALB larvae, both in laboratory and field conditions. When the release ratio of *D. helophoroides* eggs to ALB larvae was 15–25:1, the parasitism rate ranged from 60% to 100% under laboratory conditions, and releasing *D. helophoroides* in the field resulted in an ALB larval population decrease of over 86% (Li et al. 2009). Furthermore, Wei and Niu (2011) found that releasing *D. helophoroides* in the field to control ALB resulted in a substantial decrease in ALB larvae by the third year after release. The utilization efficiency of this parasitoid was further enhanced by several studies that focused on optimizing release methods, release timing, and cold tolerance of *D. helophoroides* for controlling ALB (Luo et al. 2019, Ji et al. 2021, Wen et al. 2022). In summary, *D. helophoroides* is an effective natural enemy of ALB, and its utilization for biological control represents a highly efficient and safe technique within ALB’s native range.

#### *Sclerodermus* spp.

The genus *Sclerodermus* includes over 3,000 known species worldwide. Two species (*S. guani* Xiao and Wu and *S. pupariae* Yang and Yao) are widely used in China to control young ALB larvae, which are those in the first through third instars (Luo et al. 2018, Wang 2021). These parasitoid species are effective when ALB larvae are feeding in the cambial region of their hosts (Fig. 2). While both *Sclerodermus* species prefer to parasitize young ALB larvae, parasitism rates of *S. pupariae* are significantly higher than *S. guani* (Yang et al. 2018). In augmentative releases, *S. pupariae* can reduce populations of young ALB larvae by over 62% (Kang et al. 2022). Still, *S. guani*, originally collected from *Semanotus bifasciatus* (Motschulsky) (Coleoptera: Cerambycidae) in the Sichuan Province, has been mass-reared and released as a BCA against ALB in many parts of China (Chen et al. 2003). Under laboratory conditions, *S. guani* caused average mortalities of 100%, 92%, and 87% on the first-, second-, and third-instar ALB, respectively (Yao and Yang 2008). In field experiments, *S. guani* killed 33% of early instar ALB, indicating a relatively high level of field control of young ALB larvae. As an idiobiont ectoparasitic wasp, *S. guani* feeds on and paralyzes its hosts before oviposition, causing additional host mortality. However, larger host larvae (≥3rd instar) were not as susceptible, resulting in decreased parasitism rates and direct sting-induced mortality (Yao and Yang 2008). Therefore, when using *S. guani* for controlling ALB, it is crucial to consider both the appropriate release quantity and timing to achieve optimal control effects by targeting periods when the most susceptible host larvae are the most abundant.

#### Host Specificity of *D. helophoroides* and *Sclerodermus* spp.

Both *D. helophoroides* and *Sclerodermus* spp. are generalist ectoparasitoids that can attack many woodboring beetle species. Still, the host range of *D. helophoroides* is narrow compared to *Sclerodermus*. *Dastarcus helophoroides* mainly exhibits parasitic behaviors on medium to large-sized cerambycids, including ALB, *Anoplophora chinensis* (Forster) (citrus longhorned beetle; CLB), *Apriona swainsoni* (Hope), *Batocera lineolata* Chevrolat, *Mo. alternatus*, *S. populnea*, *Xylotrechus rusticus* (Linnaeus), *Aromia bungii* (Faldermann), *Ma. raddei*, and *Mo. saltuarius* (Gebler) (Wei et al. 2013, Li 2015, Jiang et al. 2019, Wang 2020, Wen et al. 2020, Wang et al. 2021a, Zheng et al. 2022a). Field releases of *D. helophoroides* have demonstrated promising effectiveness in controlling some of these pests. For instance, when applied for control of *Mo. saltuarius*, *D. helophoroides* achieved 74% control at a parasitoid-to-pest ratio of 10:1 (Zheng et al. 2022a). In the case of *Mo. alternatus*, field releases of *D. helophoroides* adults resulted in a parasitism rate of 71% at a 1:1 parasitoid-to-pest ratio (Wu et al. 2017). Its generalist host preference enables the establishment of laboratory populations using a range of substitute hosts, reducing artificial production costs and significantly improving reproductive efficiency and utilization. As a result, *D. helophoroides* can be effectively applied for the biological control of various pest species. Still, past choice tests of various parasitoid species have demonstrated that parasitoids may prefer to attack hosts on which they developed (a phenomenon termed preimaginal learning; see Wang et al. 2019 and references therein). Nevertheless, while it does not exhibit strong host specificity, promising control effects against certain cerambycids have been achieved using *D. helophoroides*.

*Sclerodermus guani* and *S. pupariae* also exhibit low host specificity, as they can parasitize larvae and pupae of more than 50 hymenopteran, coleopteran, and lepidopteran species. In the laboratory, *Sclerodermus* species are commonly reared using substitute hosts such as the pupae of *Tenebrio molitor* Linnaeus (Coleoptera: Tenebrionidae) and larvae of both *M. alternatus* and *Thyestilla gebleri* Faldermann (Coleoptera: Cerambycidae) (Xiong et al. 2011, Gong et al. 2021, Jiang et al. 2021). Various studies have been conducted on the different hosts of these 2 parasitic wasps, revealing their potential as BCAs against pests. For instance, *S. guani* showed a favorable parasitic effect on *Apriona germari* (Hope) (Wang et al. 2020a). Tang et al. (2021) reported that both *S. guani* and *S. pupariae* can parasitize the larvae of *Alcidodes juglans* Chao (Coleoptera: Curculionidae), an insect that tunnels in small branches, which increased the potential range of hosts for biological control purposes but is worrisome for non-target species. Further, in efforts to control *M. saltuarius*, Zheng et al. (2022b) released *S. guani* and *S. pupariae* at a parasitoid-to-pest ratio of 4:1, resulting in control effects of 96% and 62%, respectively. Ren et al. (2018) observed that *S. guani* injected venom into the pupae of *T. molitor* and paralyzed hosts before oviposition. Despite these successes, it is important to note that the broad host range and lower host specificity of *Sclerodermus* wasps can produce significant impacts on non-target hosts when released in the field. Careful consideration and assessment of potential non-target effects are necessary when utilizing these parasitic wasps for pest control purposes. In summary, *S. guani* and *S. pupariae* show promising results as BCAs against some woodboring pests such as *A. germari* and *M. saltuarius*, but they can parasitize a wide range of non-target insect species.

As both *D. helophoroides* and *Sclerodermus* spp. are generalist ectoparasitoids, these BCAs are not recommended for ALB management outside of Asia due to potential non-target risks to woodboring beetle communities in invaded areas (Smith et al. 2007, Gould et al. 2018, Kim et al. 2018, Rim et al. 2018). Thus, host-specific parasitoids are necessary for biological control outside of ALB’s native range.

#### The Search for Host-specific Parasitoids

**Fig. 3.**
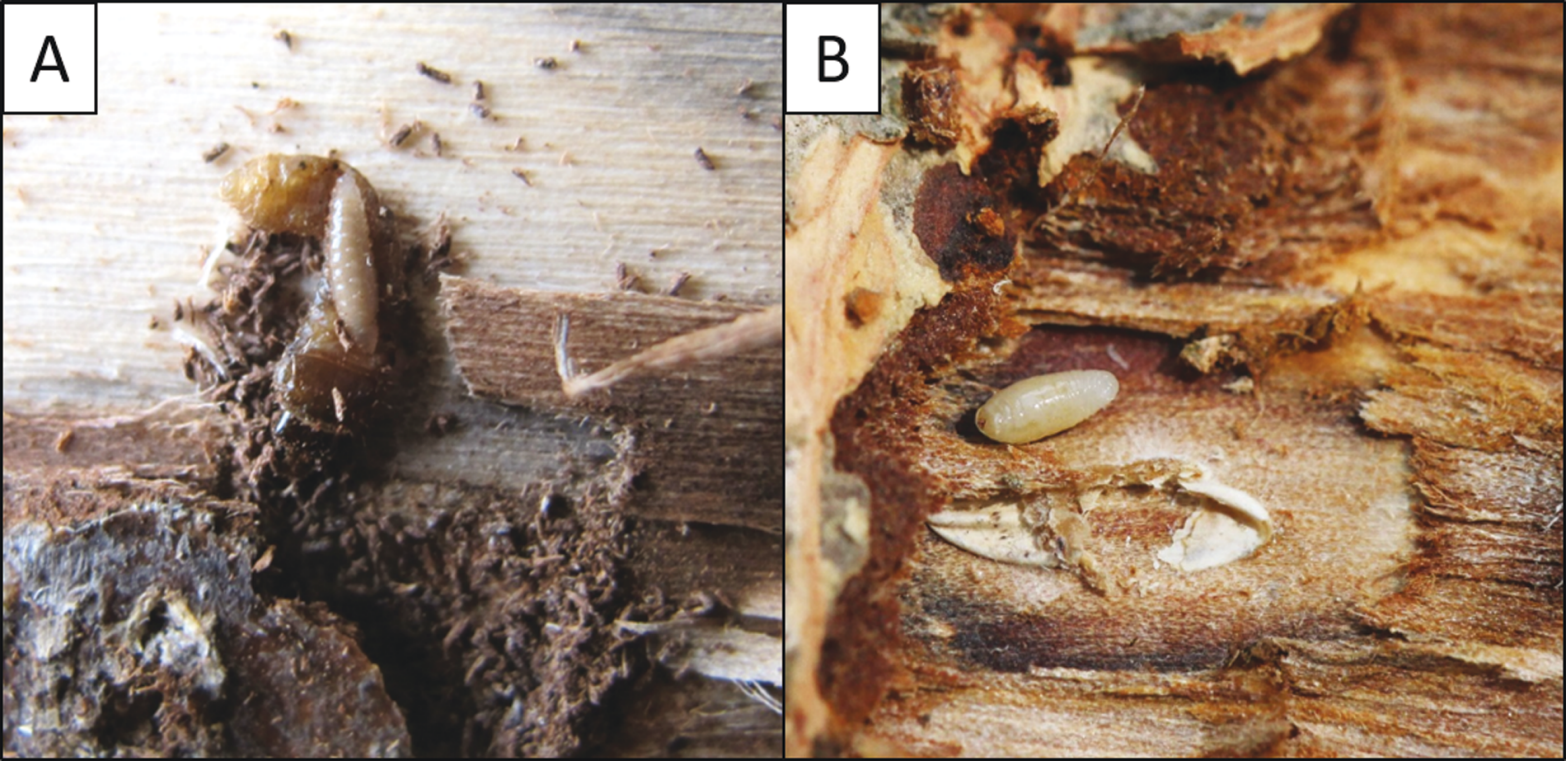
Immature stages of a larval parasitoid, *Bracon planitibiae* (A), and an unidentified putative egg parasitoid from Lee et al. 2023 (B). Photo credits: (A) Liang-Ming Cao, Ecology and Nature Conservation Institute, Chinese Academy of Forestry; (B) Seunghyun Lee, Natural History Museum London.

Recently, sentinel log traps have been utilized for survey efforts in an attempt to find host-specific BCAs for ALB. A sentinel log trap is a method employed to monitor or capture specific woodboring insects and their associated natural enemies. It involves placing logs or wood pieces, usually from potential host tree species, in designated areas suspected to be habitats of the target insect (Mansfield et al. 2019). The sentinel log traps contain susceptible hosts in known quantities and locations, and after being placed in the field for a set period of time, are harvested and destructively dissected to determine if any of the hosts were parasitized. Using this method, explorations in South Korea found a larval parasitoid, *Leluthia honshuensis* Belokobylskij and Maeto (Hymenoptera: Braconidae), attacking early instar ALB (Kim et al. 2018). Additional ALB parasitoid surveys in South Korea exclusively aimed to discover egg parasitoids using a new sentinel log trap method. In these surveys, ALB adults and sentinel log traps were placed into mesh cages near existing oviposition pits, which allowed ALB to oviposit on the sentinel log traps in the field (Lee et al. 2023). These studies encompassed over 400 logs and 3,000 ALB eggs in both central (Gapyeong) and southern (Busan) South Korea between 2019 and 2022. The sentinel log traps led to the discovery of a potential ectoparasitoid of ALB eggs (Fig. 3B). However, individuals perished during pupation and morphological and molecular identification attempts failed, resulting in uncertain classification at the family level. Additionally, another early larval stage ALB parasitoid, *Spathius ibarakius* Belokobylskij and Maeto, was collected during this study, but as the study was tailored to detect egg parasitoids, the sentinel logs were swiftly collected, leaving parasitoids of later instars undiscovered (Lee et al. 2023).

**Fig. 4.**
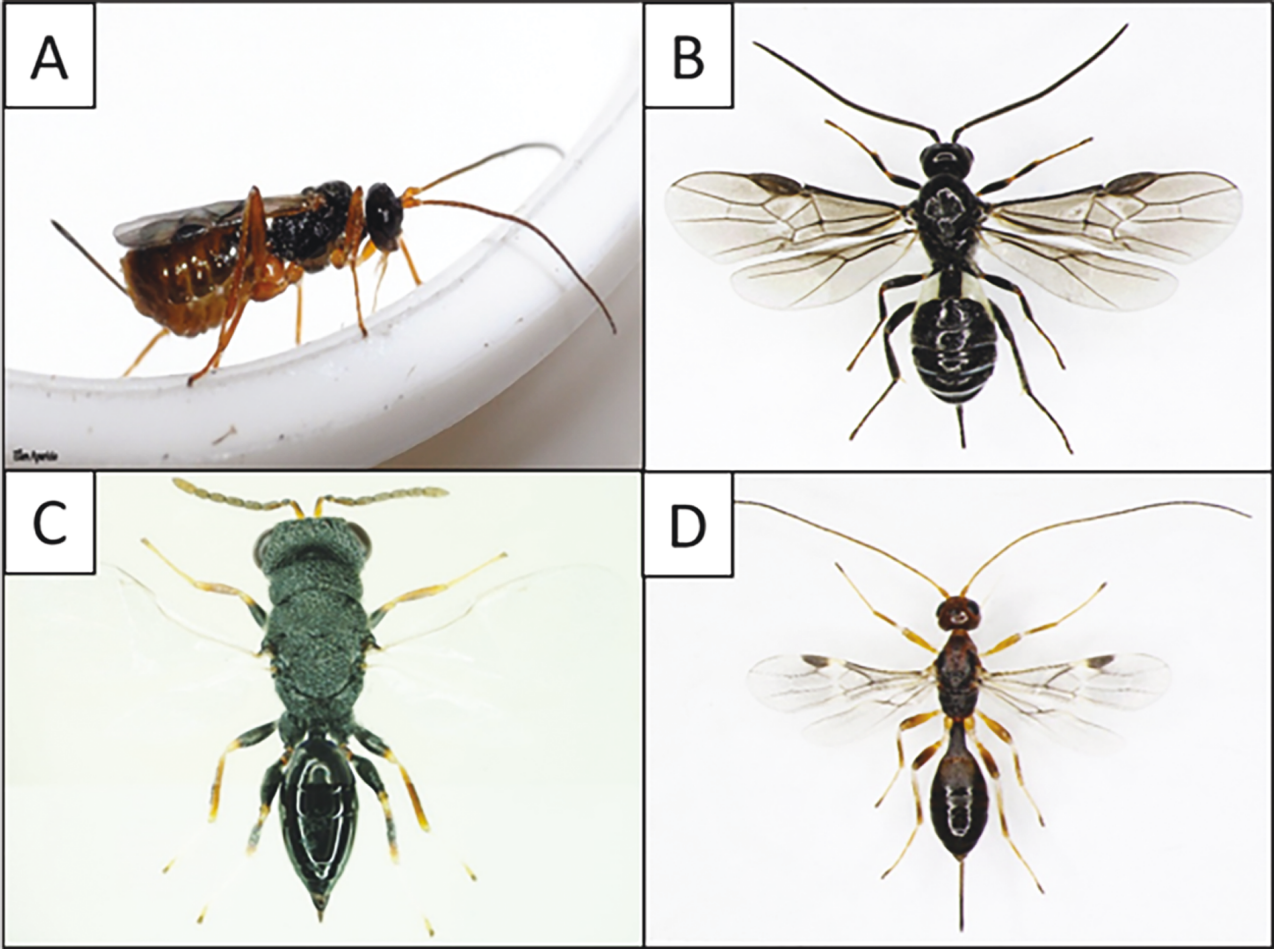
Explorations and laboratory tests have uncovered several parasitoids capable of attacking ALB in both its native and invaded ranges. These include *Ontsira mellipes* (A*), Bracon plantibiae* (B), *Eurytoma morio* (C), and *Spathius anoplophorae* (D). Photo credit: (A) Ellen Aparicio, Agricultural Research Service, USDA; (B), (C), (D) Liang-Ming Cao, Ecology and Nature Conservation Institute, Chinese Academy of Forestry

In addition to the species found during surveys in South Korea, sentinel log surveys have been conducted extensively from 2015 to 2022 in 6 different geographical regions in China, discovering several potential BCAs (Li et al. 2020, Wei et al. 2023). One early survey in China indicated 2 possible egg parasitoid species, *Xorides* sp. (Hymenoptera: Ichneumonidae) and *Anastatus* sp. (Hymenoptera: Eupelmidae) (Li et al. 2020). However, all known species in the genus *Xorides* are larval ectoparasitoids (see Wang et al. 2021c), and the species collected in the genus *Anastatus* was found only once in Shanghai with extremely low parasitism (0.8%). Furthermore, the *Anastatus* sp. was not found again in the later surveys at the same location (Wei et al. 2023). As such, whether *Xorides* sp. or *Anastatus* sp. are ALB egg parasitoids remains to be confirmed. Past surveys using destructive sampling (i.e., felling and dissecting infested trees) failed to recover ALB egg parasitoids in China (Liu et al. 2016, Golec et al. 2018). In total, 12 parasitic natural enemies were discovered from the recent sentinel log surveys in China (Table 2). However, most of the species were rare or had very low parasitism, and most parasitoid species are not found ubiquitously across regions. For example, among these parasitoids, 8 species were found in both Beijing and Shanghai, while only one species was discovered in Hunchun, Jilin Province. From 2019 to 2022, Wei et al. (2023) discovered 7 species of parasitoids of early instar ALB larvae in Beijing, Shanghai, Tianshui (Gansu Province), Kunming (Yunnan Province), and Zunyi City (Guizhou Province) (Table 2). All 7 species of parasitic wasps were found in Beijing, 4 were found in Shanghai, 3 were found in Zunyi, 2 were found in Kunming, and none were found in Tianshui. While several species (e.g., *Bracon planitibiae*, Figs. 3A and 4B) were discovered in both studies, the larval parasitoids *Spathius anoplophorae* Yang (Fig. 4D) and *Oxysychus glabripennis* Yang (Hymenoptera: Pteromalidae) were commonly collected in different regions (Li et al. 2020, Wei et al. 2023).

Due to their consistent discovery, both *S. anoplophorae* and *O. glabripennis* were imported into the USDA Agricultural Research Service Beneficial Insects Introduction Research Unit (BIIRU) for quarantine evaluations. *Spathius anoplophorae* has been successfully reared on ALB logs at BIIRU for over 30 generations from 2022 to 2024 (Wang et al. unpublished data). This parasitoid has also been successfully reared at the Chinese Academy of Forestry in China (Li et al. 2020). On average, it developed from egg to adult from 11.1 to 22.3 days at 21 to 33°C, and each female produced about 30 offspring, which were female-biased (80%) (Li 2020). The female-biased progeny sex ratio and short developmental time are important attributes of a promising BCA (Heimpel and Lundgren 2000). However, host specificity tests in China showed that *S. anoplophorae* was able to parasitize several other non-target species, including CLB, *A. bungii*, *M. alternatus*, *Eucryptorrhynchus brandti* (Harold) (Coleoptera: Curculionidae), and *A. planipennis*, but it did not attack *A. germari* or *T. gebleri* (Li 2020). In the United States, host specificity tests also showed that the parasitoid could attack CLB along with the common North American cerambycid *Monochamus scutellatus* (Say) and, to a lesser degree, *A. planipennis*, but it did not attack *Xylotrechus colonus* (Fabricius) (Coleoptera: Cerambycidae) (Wang et al. unpublished data). These studies suggest that *S. anoplophorae* has the potential to attack some common non-target woodboring beetles infesting conifers and hardwoods in China or North America, especially from the same subfamily (Lamiinae) as ALB. Little is known about the biology of the other newly discovered Asian parasitoids, but the other 2 *Spathius* spp. recorded from South Korea were also known to attack related cerambycids (Kim et al. 2018, Lee et al. 2023).

### Potential for Novel-association Biological Control: Parasitoids of ALB in North America and Europe

Recent studies identified several native North American parasitoids that could serve as novel-association BCAs, as these cerambycid parasitoids can attack ALB larvae in laboratory tests. Golec et al. (2020) conducted extensive surveys of cerambycid parasitoids in Mid-Atlantic forests in the United States Over a 7-yr survey, more than 14,500 cerambycids from 56 species and 38 genera were collected, and more than 19,000 parasitic hymenopterans from 12 families emerged from woodborer infested material. Over 70% of individuals belonged to the family Braconidae, including 13 known and 2 unknown species. Among them were *Ontsira mellipes* Ashmead (Fig. 4A) and *Rhoptrocentrus piceus* Marshall, which accounted for 53.1% and 23.8% of parasitoids that emerged, respectively. These and several other braconids (including *Spathius laflammei* Provancher and 2 unidentified *Heterospilus* and *Atanycolus* species) were found to be capable of attacking ALB in laboratory tests (Duan et al. 2016). These parasitoids are currently being evaluated for the potential use as novel-association BCAs against ALB in recently quarantined areas.

Similarly, several native European parasitoids of woodborers were found to parasitize ALB and/or CLB larvae. There include *Spathius erythrocephalus* Wesmael, *Eurytoma melanoneura* Walker (Hymenoptera: Eurytomidae), and *E. morio* Boheman (Fig. 4C); *Calosota vernalis* Curtis (Hymenoptera: Eupelmidae); *Cleonymus brevis* Boucek (Hymenoptera: Cleonymidae); *Trigonoderus princeps* Westwood (Hymenoptera: Pteromalidae); and *Sclerodermus* spp. in Italy (Hérard et al. 2013, Brabbs et al. 2014). *Sclerodermus brevicornis* (Kieffer), a native European parasitoid, could readily attack ALB, CLB, and *Psacothea hilaris* (Pascoe) (Coleoptera: Cerambycidae) in a laboratory test (Lupi et al. 2017) (Table 2), making it a potential novel-association BCA.

All the resident parasitoids reported in the United States and Europe are generalist larval ectoparasitoids (Table 2) that seem to have the potential to adapt to ALB via novel associations. Most of these have been recorded as ectoparasitoids of woodboring beetle larvae in the families Buprestidae, Cerambycidae, and Curculionidae: Scolytinae. For example, *Atanycolus* spp. and *S. laflammei* have been reported attacking EAB in the field (Duan et al. 2013) and ALB in the laboratory (Duan et al. 2016), whereas *Heterospilus* spp. are reported primarily attacking bark beetles in the subfamily Scolytinae (Wegensteiner et al. 2015). Furthermore, *R. piceus* has been reported from cerambycids in 6 genera (Golec et al. 2020). With the exception of *O. mellipes*, there is very little information about the biology, life cycle, and host ranges of these resident parasitoids capable of attacking ALB in the United States or Europe (Table 2).

#### Ontsira mellipes

Various aspects of the biology of *O. mellipes* associated with ALB, including life history, larval development, fecundity, host specificity, and rearing methods, have been investigated (Duan et al. 2016, Golec et al. 2016, 2017, Wang et al. 2019, 2020b, Wang and Aparicio 2020). These studies showed that female *O. mellipes* emerged with a substantial portion (38%) of their lifetime complement of mature eggs. Additionally, these eggs matured rapidly, sometimes as quickly as 4–6 days post-eclosion. The number of mature eggs was positively correlated to the female wasp’s body size and oviposition by young wasps prompted the production of more mature eggs (Wang and Aparicio 2020). A female *O. mellipes* paralyzes the host larva prior to laying a clutch of eggs on the surface of the host, and clutch size increases with increasing host size (Golec et al. 2016, Wang and Aparicio 2020). At 23°C, the parasitoid eggs hatch in 2–3 days, larvae develop in about 8–10 days and pupate adjacent to the consumed host, and adults emerge in about 10–15 days (Golec et al. 2016). Parasitoid offspring are female-biased (85% female), and each female can produce up to an average of 6.8 female progeny per ALB larva and a total of 21.9 progeny per wasp (Wang and Aparicio 2020). Recent evaluations also showed that parasitism on ALB increased with later generations (Golec et al. 2019). Host size did not affect the parasitoid’s offspring survival, developmental time, or sex ratio, but female wasps that developed from large hosts had larger body size and consequently a higher mature egg load than those reared from small hosts (Wang and Aparicio 2020). Host specificity testing in the laboratory showed that *O. mellipes* would attack 3 of the 6 tested North American cerambycid species: *Elaphidion mucronatum* (Say), *Monochamus carolinensis* Olivier, and *M. notatus* (Drury), but it would not attack *Neoclytus scutellaris* Olivier*, Xylotrechus colonus,* or *X. sagittatus* (Germar) (Wang et al. 2019). Under natural conditions, *O. mellipes* may be unable to reach certain host larvae that are feeding deep within the wood due to the limited length of the female wasp’s ovipositor. It is also important to note that these attacked cerambycids occurred across hardwoods and conifers, meaning *O. mellipes* could potentially impact a wider range of organisms if distributed more broadly. Overall, parasitism and total numbers of parasitized hosts or progeny produced increased with host and/or parasitoid densities, and superparasitism did not occur, likely because attacked hosts are no longer detectable (Wang et al. 2020b). Per capita parasitization efficiency or reproductive outcomes were optimized at a low parasitoid-host ratio but with large group size of hosts and parasitoids. For efficient mass-rearing of the parasitoid, an optimal combination of exposing 3 or 4 parasitoids to 4 hosts is proposed (Wang et al. 2020b).

In conclusion, there is a potential to use natural enemies native to the pest’s introduced regions, such as *O. mellipes*, via novel associations for biological control of ALB. These native parasitoids may also be mass reared for augmentative releases to help with the eradication efforts of ALB. However, the efficiency of such novel-association biological control has not yet been demonstrated in the field.

## Feasibility of Biological Control Programs Against ALB

Historically, the management of ALB in China has primarily relied on traditional chemical insecticides. While this method is effective, it may not be feasible in all scenarios and can lead to environmental contamination and biodiversity loss due to non-target impacts of the pesticides. Therefore, biological control is regarded as a preferred choice for ALB management in China, and the primary BCAs used are *D. helophoroides* and *Sclerodermus* spp. (Liang et al. 2013).

However, in Korea, neither chemical nor biological control is typically implemented for ALB. Here, the extent of ALB’s damage is largely unknown, and it was believed that only native populations existed. However, a recent study revealed the coexistence of both native and invasive ALB populations in South Korea (Lee et al. 2020). The invasive populations were first observed in large port cities, and individuals were collected on both native and imported street trees. Generally, invasive populations cause more severe damage than native populations (Williams et al. 2004, Lee et al. 2020). The native ALB population is distributed in the northeast to the central part of South Korea, and severe damage within this range only occurs on certain planted *Acer* spp. (Lee et al. 2023). In such cases, simply removing planted trees effectively decreases the population (personal observation, Seunghyun Lee), potentially being more effective than using biological control. Therefore, due to the challenge of determining the predominant cerambycid presence in natural forests and accurately assessing their impact on coexisting species, the utilization of BCAs should be approached with the utmost caution.

Conversely, ALB damage is more severe in urban areas of Korea where invasive populations are found in southern and western cities, and their range seems to be expanding (Lee et al. 2020). In these areas, the extremely high population levels of ALB could warrant the implementation of biological control. However, different biological control tactics must be established depending on the circumstances of each region. For example, the BCAs may not need to be entirely host-specific in some regions. In Seoul, invasive populations of ALB reside in extremely urbanized landscapes and rarely cohabit with other cerambycids that have similar size or larval feeding habits. Under such circumstances, the utilization of native BCAs like *S. ibarakius*, despite its attack on at least 2 host species, could be considered. In contrast, in southern cities where ALB populations exist, numerous forests in close proximity suggest potential habitat overlap with various native cerambycid species. The artificial release of generalist BCAs in these regions might inadvertently affect these non-target species. Therefore, a cautious approach is needed in these areas.

Experiments utilizing sentinel logs in Korea and China have highlighted the difficulties in detecting egg parasitoids of ALB, even within their native environments (Kim et al. 2018, Li et al. 2020, Lee et al. 2023, Wei et al. 2023). While egg parasitoids are generally considered efficient BCAs because they parasitize pests before they cause damage (Mills 2010), few egg parasitoids have been reported on woodboring cerambycids, and the majority (>80%) of known cerambycid parasitoids worldwide are larval ectoparasitoids (Wang et al. 2021c). One plausible explanation for the lack of egg parasitoids of ALB may be the cryptic nature of ALB eggs (Wang et al. 2021c). Female ALB oviposits by chewing through the full depth of the bark, and the egg is placed under the bark in the cambial region and covered with a plug, which seems to physically prevent egg parasitoids from accessing ALB eggs (Wang et al. 2021c, Wei et al. 2023). In contrast, most cerambycids lay eggs on bark surfaces or in bark cracks or crevices (Hanks and Wang 2017). For example, *P. semipunctata*, which lays eggs in bark cracks, is successfully controlled by the egg parasitoid *Avetianella longoi* Siscaro (Hymenoptera: Encyrtidae), introduced from Australia into California, becoming one of the only successful examples of classical biological control of cerambycids (Hanks et al. 1996). Females of some lamiine species such as ALB and CLB insert eggs into bark. While CLB inserts its egg into the bark, it differs from ALB in that the bark often cracks, partially exposing the egg (Haack et al. 2010). As such, the egg parastioid *Aprostocetus fukutai* Miwa and Sonan (Hymenoptera: Eulophidae) is able to locate CLB eggs through this crack (Wang et al. 2021b, 2022).

Generally, egg parasitoids are used for hemipteran or lepidopteran pests (Parra et al. 1987, Mills 2010, de Almeida et al. 2015), which lay eggs externally on host plants; these pests do not damage the plants until the larvae or nymphs emerge and feed. In contrast, ALB causes minor damage during oviposition, chewing pits, and depositing eggs in the cambial region of the host. Therefore, larval parasitoids that target early ALB instars might offer a similar level of effectiveness as egg parasitoids. Consequently, the future focus should lean toward identifying early-stage parasitic wasps rather than egg parasitoids.

### Feasibility of Biological Control in the Invaded Area of ALB

The invaded areas of ALB align with the latitudinal range of its native habitat, suggesting climates may be suitable for introducing natural enemies from its native range. While *S. anoplophorae* has been successfully reared in the laboratory, further studies are needed to determine its host specificity and potential as a BCA in invaded areas. As previously discussed, *D. helophoroides* and *Sclerodermus* spp. play a crucial role in the effective control of ALB in China, but their wide host range may pose unexpected ecological risks to local ecosystems in ALB’s invaded range (Howarth 1991, Simberloff et al. 1996, Hajek et al. 2016).

Augmentative releases of novel-association BCAs remain an option for ALB management and may be used in conjunction with classical biological control. For example, one study demonstrated that the larval density of EAB was initially reduced by native parasitoids (including *Atanycolus* spp.) and then by an introduced BCA in Michigan, United States (Duan et al. 2015). Identifying and testing native BCAs in the invaded areas of ALB could provide insight into this method. *Onstira mellipes* is a promising BCA, but field research is lacking. More information is needed on the biology, life cycle, and efficacy as a BCA of this and other North American parasitoids as well as those discovered in Europe.

### Challenges in Biological Control of ALB

Throughout this paper, we have discussed several challenges to be addressed before the successful implementation of ALB biological control in invaded areas. Many of these are specific to ALB, for example, the generalist feeding behaviors of some BCAs emphasize the importance of exploring more host-specific BCAs. Additionally, rearing methods have not been developed for all parasitoids and when developed, can be costly, time consuming, and labor intensive. The cost of rearing ALB on its own is significant, estimated to be about $21 USD per beetle in 2005 (Keena 2005). Due to the expenses and challenges associated with rearing ALB and its parasitoids, it is important to have a strong understanding of the suitable site and environmental characteristics necessary for release and establishment success. Such release guidelines are emphasized in other biological control programs, such as EAB (USDA-APHIS/ARS/FS 2024). Additionally, it is important to understand the life cycle and phenology of parasitoids and their hosts to ensure biological and phenological synchronization, particularly in novel environments. For instance, if adult egg parasitoids *A. longoi* were active in winter, they would not find host eggs because *P. semipunctata* is inactive at this time (Hanks et al. 1996). Most BCAs mentioned here attack the young larval stages of ALB and would require adult releases to occur when these ALB stages are present. Both the phenology and biological control of ALB have been explored in the novel environment of South Carolina, United States to address this challenge (Lupu 2024, Schmitt et al. 2024a, 2024b), and opportunities for similar research exist throughout ALB’s range to optimize releases of BCAs.

## Conclusion

We are still a long way from the development of a successful biological control program against ALB due to the difficulty in studying ALB and its natural enemy population dynamics in both its native and invaded ranges as well as limited long-term financial support for the effort (relative to other strategies such as eradication). It is still debatable whether this is a suitable management strategy against ALB at all. Successful eradication of ALB has been achieved in about half of the invasive populations and remains the goal of most management programs. As biological control programs are typically implemented in cases where pests are established and management objectives are to reduce pest populations and long-term damage to host species, the notion of a long-term ALB population and management strategy flies in the face of current efforts. However, some conditions may support biological control as a complement to host removal to achieve eradication. For example, in the ALB infestation in South Carolina, United States, the swampy landscapes prohibit access by heavy machinery. Here, biological control may act to supplement host removal to achieve eradication. If management program objectives ever pivot from eradication to population reduction, much of the groundwork will have been accomplished to implement biological control. Thus, these factors suggest the pursuit of BCAs against ALB is a worthwhile endeavor, despite the challenges.

## Data Availability

Data underlying the article are available in the article.
